# Supplementary material for: Label-free biosensor assay decodes the dynamics of Toll-like receptor signaling
Source: Nat Commun. 2024 Nov 12;15:9554. doi: 10.1038/s41467-024-53770-9 (PMC11558003; doi:10.1038/s41467-024-53770-9)
Supplement: Supplementary file 1 — Supplementary Information [file 41467_2024_53770_MOESM1_ESM.pdf]

Supplementary Information

Label-free biosensor assay decodes the dynamics of Toll-like receptor signaling

Janine Holze[1], Felicitas Lauber[1], Sofía Soler[2], Evi Kostenis[3], Günther Weindl[1,*]

[1]Pharmaceutical Institute, Section Pharmacology and Toxicology, University of Bonn, Bonn, Germany

[2]Institute of Experimental Haematology and Transfusion Medicine, University Hospital Bonn, Bonn, Germany.

[3]Institute for Pharmaceutical Biology, Molecular, Cellular and Pharmacobiology Section, University of Bonn, Bonn, Germany

[*]e-mail: guenther.weindl@uni-bonn.de

This PDF file includes:

Supplementary Figures 1 to 16

Uncropped images of Western blots in Supplementary Figure 16

Supplemental References

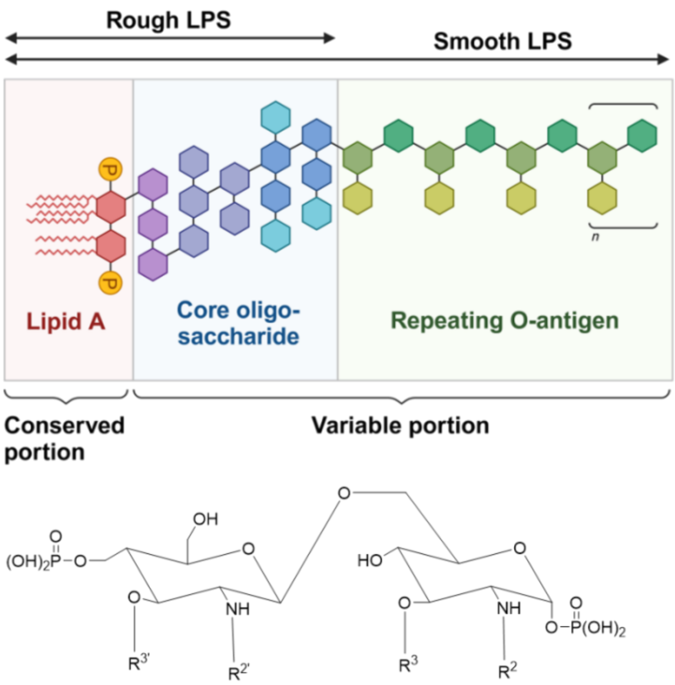

Bacterial species	$R^{3'}$	$R^{2'}$	R^3	R^2	Reference
S. minnesota	C_{14}-O-(C_{14})	C_{14}-O-(C_{12})	C_{14}-OH	C_{14}-O-(C_{16})	[1]
E. coli	C_{14}-O-(C_{14})	C_{14}-O-(C_{12})	C_{14}-OH	C_{14}-OH	[2]
R. sphaeroides	C_{10}-OH	C_{14}-O-(C_{14}:1 Δ7)	C_{10}-OH	C_{14}-OH	[3]

Supplementary Figure 1. **Schematic structures of LPS.** In the scheme, lipid A structures from LPS of *E. coli*[1], *S. minnesota*[2] and *R. sphaeroides*[3] specifying the number, length, and type of acyl chains for each lipid A variant are presented. Adapted from Alexander et al[4]. Scheme created in BioRender. Weindl, G. (2024) BioRender.com/b37l145

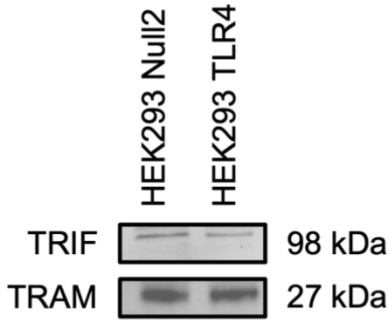

Supplementary Figure 2. HEK293 cells express adaptor proteins TRIF and TRAM. Western blot analysis of the expression levels of TRIF and TRAM in control HEK293 cells (HEK293 Null2) and HEK293 TLR4/MD-2/CD14 cells (HEK293 TLR4). Data are representative of three biologically independent experiments. Uncropped versions of blots are provided in Supplementary Fig. 16.

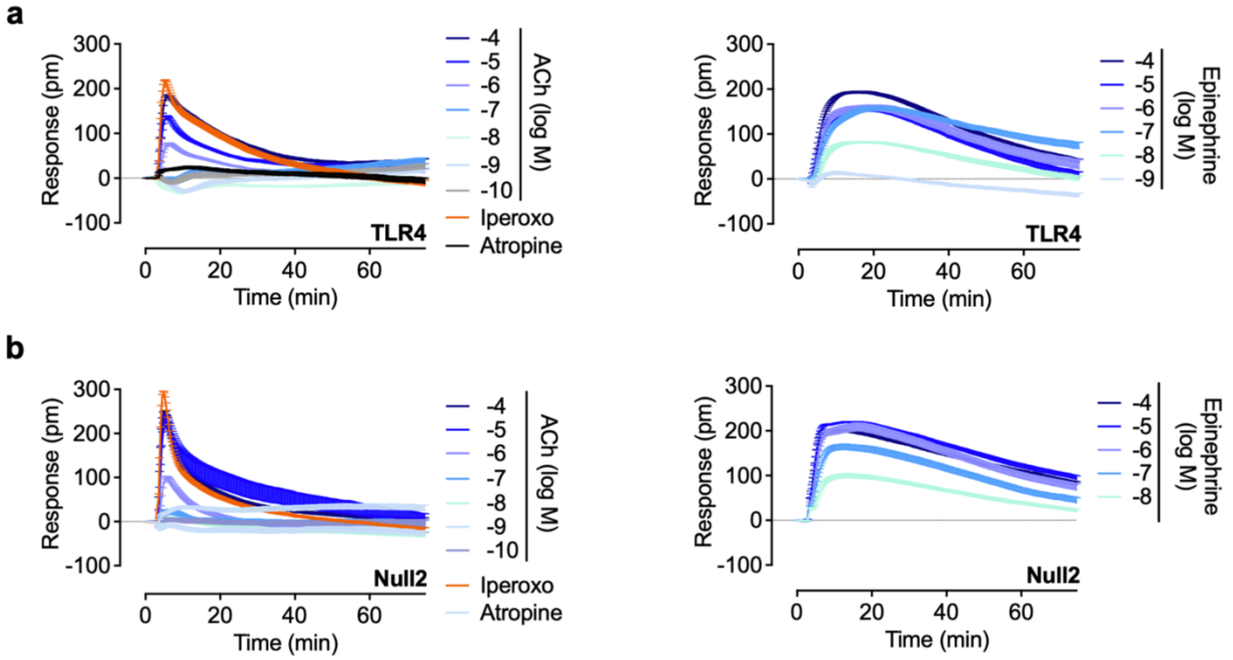

Supplementary Figure 3. Optical biosensor shows consistent GPCR signaling patterns in HEK293 cells. a, **b** HEK293 TLR4/MD-2/CD14 reporter cells (**a**) or control HEK293 cells (Null2) (**b**) stimulated with the indicated concentrations of acetylcholine (ACh) and epinephrine, iperoxo (100 µM), and atropine (10 µM). ACh visualizes signaling along the $G_{q\alpha/11}$ pathway whereas epinephrine induces presumably the G_s pathway. Baseline-corrected DMR recordings are mean + SEM and representative of three biologically independent experiments. Source data are provided as a Source Data file.

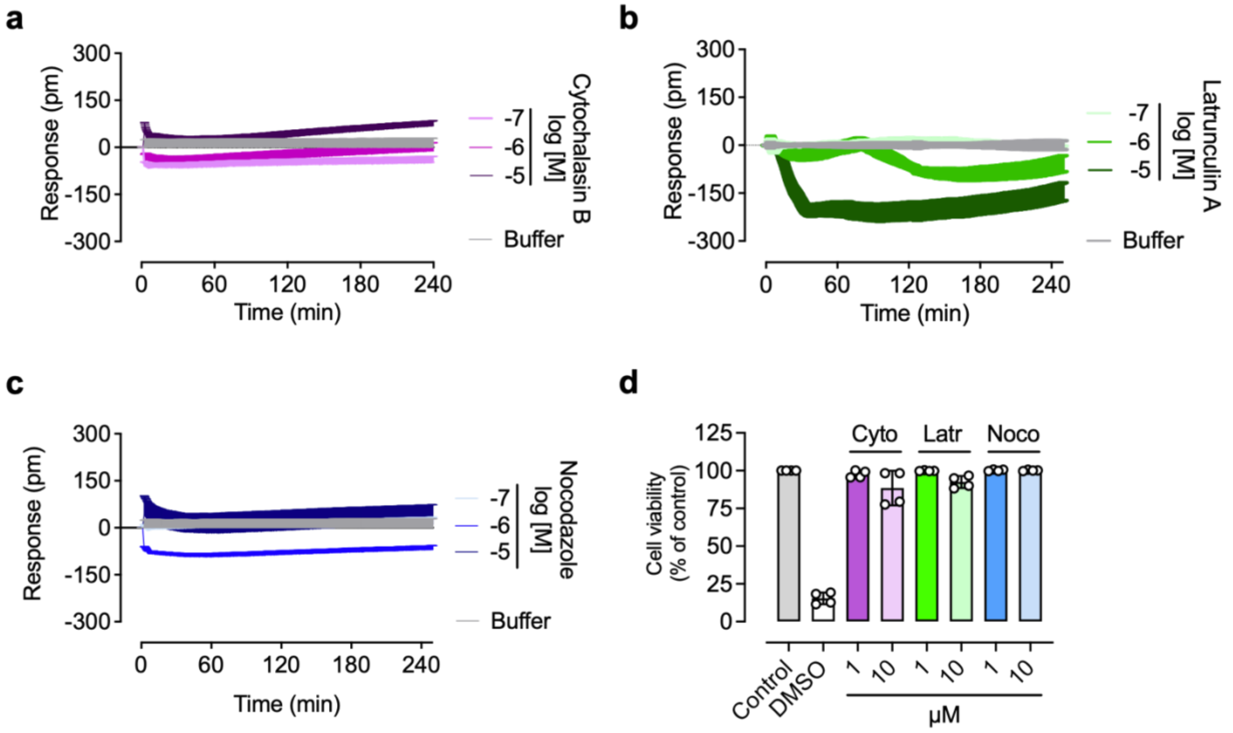

Supplementary Figure 4. Actin and tubulin inhibitors do not affect cell viability. **a**-**c** HEK293 TLR4/MD-2/CD14 reporter cells were stimulated with the indicated concentrations (log M) of the actin inhibitors cytochalasin B (**a**) and latrunculin A (**b**), as well as the tubulin inhibitor nocodazole (**c**). Baseline-corrected DMR recordings are mean + SEM and representative of three biologically independent experiments. **d** HEK293 TLR4/MD-2/CD14 cells were incubated with the actin and tubulin inhibitors for 4 h. Cell viability was determined by MTT assay and normalized to non-stimulated cells. DMSO (30%, v/v) served as control. Values presented are mean ± SEM (*n* = 4 biologically independent experiments). Source data are provided as a Source Data file.

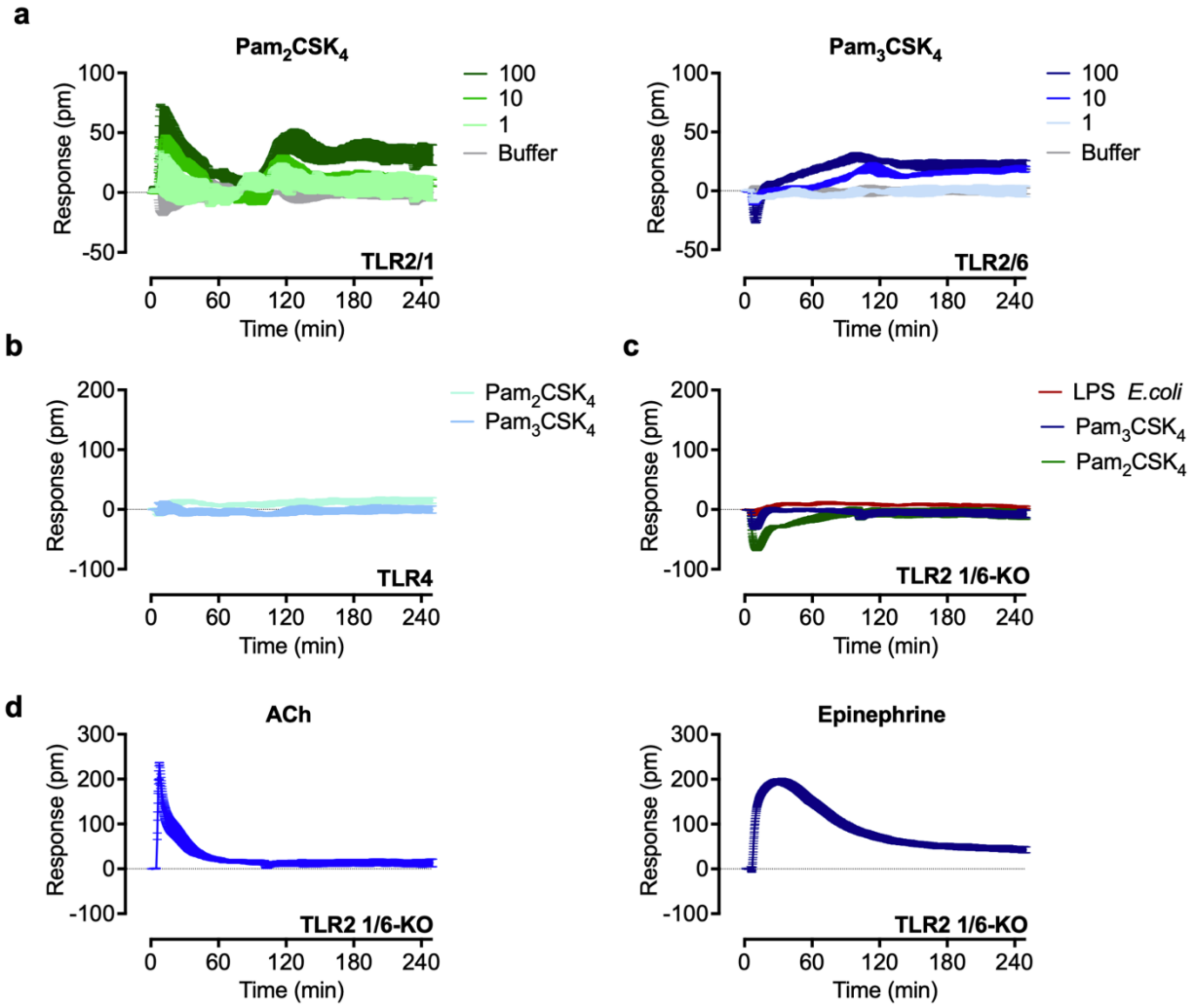

Supplementary Figure 5. DMR responses induced by Pam_3CSK_4 and Pam_2CSK_4 in HEK293 cells. **a** HEK293 TLR2/1 or 2/6 reporter cells stimulated with the indicated concentrations (ng/ml) of the TLR2 agonists Pam_2CSK_4 and Pam_3CSK_4. **b** HEK293 TLR4/MD-2/CD14 reporter cells stimulated with the TLR2 agonists Pam_2CSK_4 (100 ng/ml) and Pam_3CSK_4 (100 ng/ml). **c, d** HEK293 TLR2 1/6-KO cells stimulated with the TLR4 agonist LPS *E.coli* (10 ng/ml), TLR2/1 agonist Pam_3CSK_4 (100 ng/ml), TLR2/6 agonist Pam_2CSK_4 (100 ng/ml) (**c**), acetylcholine (100 µM) or epinephrine (100 µM) (**d**). Baseline-corrected DMR recordings are mean + SEM and representative of three biologically independent experiments. Source data are provided as a Source Data file.

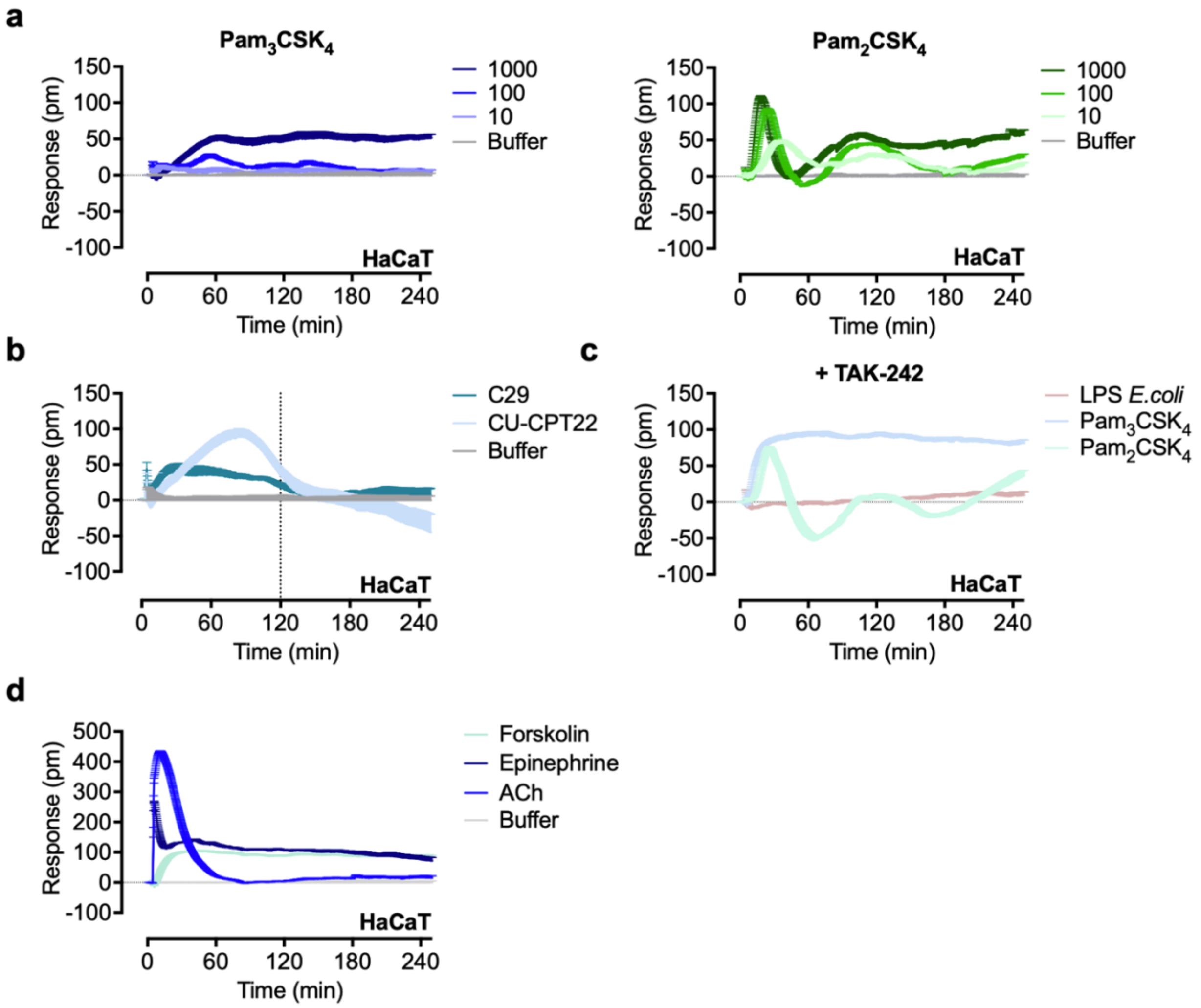

Supplementary Figure 6. Optical biosensor assay in HaCaT cells. a HaCaT cells stimulated with the indicated concentrations (ng/ml) of TLR2/6 agonist Pam_2CSK_4 or TLR2/1 agonist Pam_3CSK_4. **b** HaCaT cells incubated with the TLR2 antagonists CU-CPT22 (50 µM) and C29 (50 µM). The dashed line indicates the preincubation time (120 min) of the TLR2 antagonists. **c** HaCaT cells stimulated with the TLR4 agonist LPS *E. coli* (100 ng/ml), TLR2/1 agonist Pam_3CSK_4 (100 ng/ml) or TLR2/6 agonist Pam_2CSK_4 (100 ng/ml) preincubated with the TLR4 inhibitor TAK-242 (50 µM). **d** HaCaT cells stimulated with ACh (100 µM), epinephrine (100 µM) and forskolin (10 µM). Baseline-corrected DMR recordings are mean + SEM and representative of three biologically independent experiments. Source data are provided as a Source Data file.

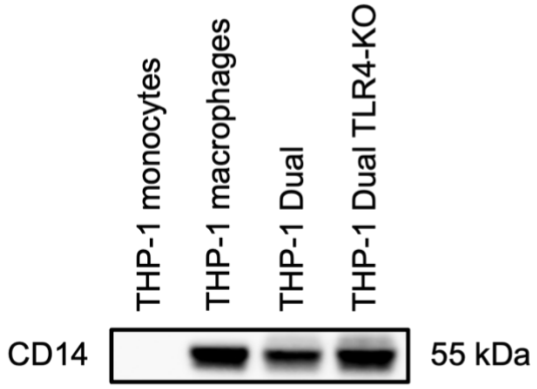

Supplementary Figure 7. THP-1 monocytes express no CD14. Western blot analysis of CD14 expression levels in THP-1 monocytes, PMA-differentiated THP-1 macrophages, THP-1 TLR4/MD-2/CD14 (THP-1 Dual) and THP-1 Dual KO-TLR4/MD-2/CD14 (THP-1 Dual TLR4-KO) cells. Data are representative of three biologically independent experiments. Uncropped version of blot is provided in Supplementary Fig. 16.

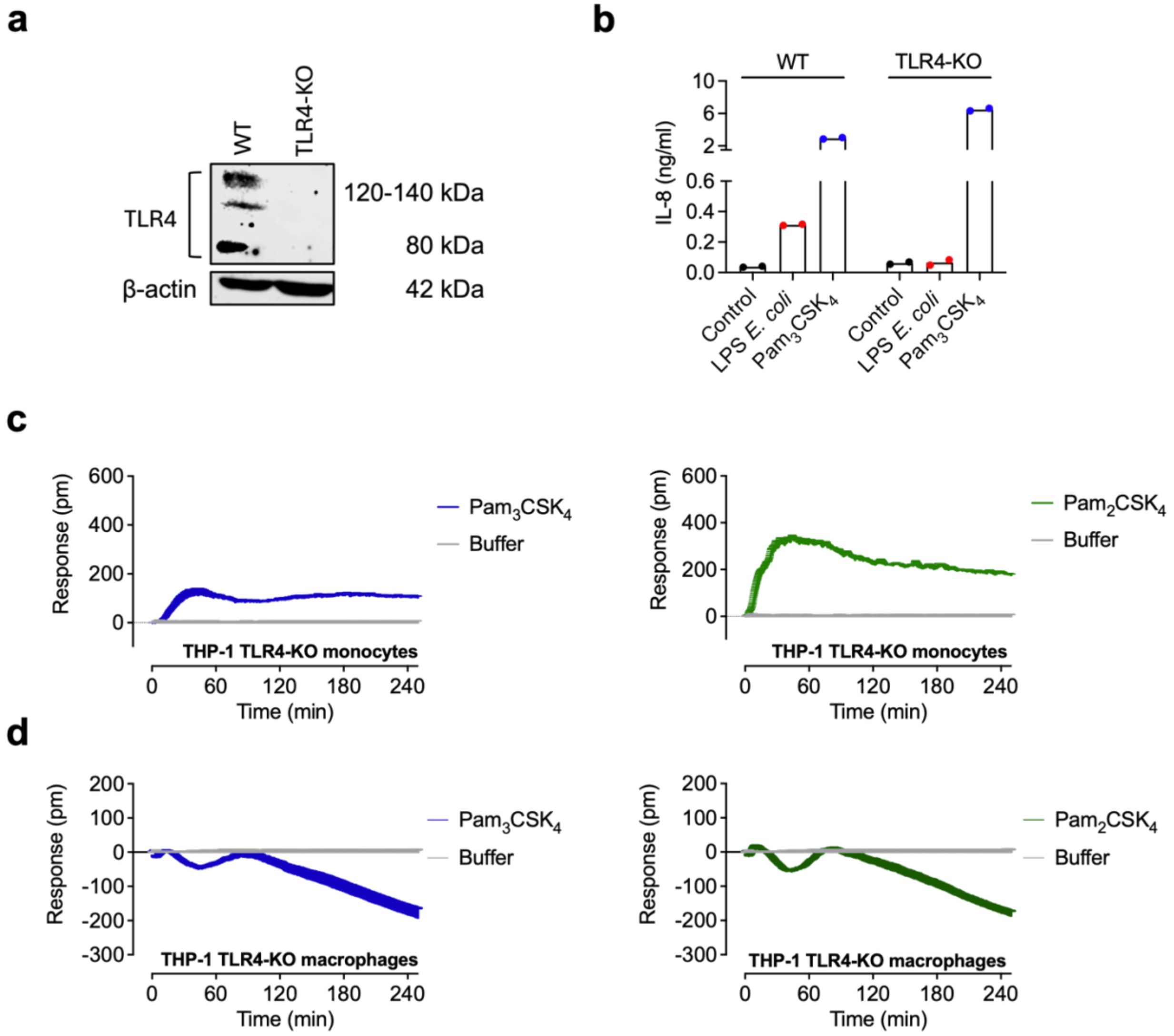

Supplementary Figure 8. THP-1 TLR4-KO cells do not respond to LPS but TLR2 ligands. a Western blot analysis of TLR4 expression in THP-1 WT and TLR4-KO cells. β-actin served as an internal control. Data are representative of two biologically independent experiments. Uncropped version of blot is provided in Supplementary Fig. 16. **b** THP-1 WT or TLR4-KO macrophages were stimulated with LPS *E. coli* (1 µg/ml) or Pam_3CSK_4 (500 ng/ml) overnight. IL-8 release into the cell-free supernatants was determined by ELISA (*n* = 2 biological replicates). **c, d** THP-1 TLR4-KO monocytes (**c**) or macrophages (**d**) were stimulated with Pam_3CSK_4 (1000 ng/ml) and Pam_2CSK_4 (1000 ng/ml). Baseline-corrected DMR recordings are mean + SEM and representative of three biologically independent experiments. Source data are provided as a Source Data file.

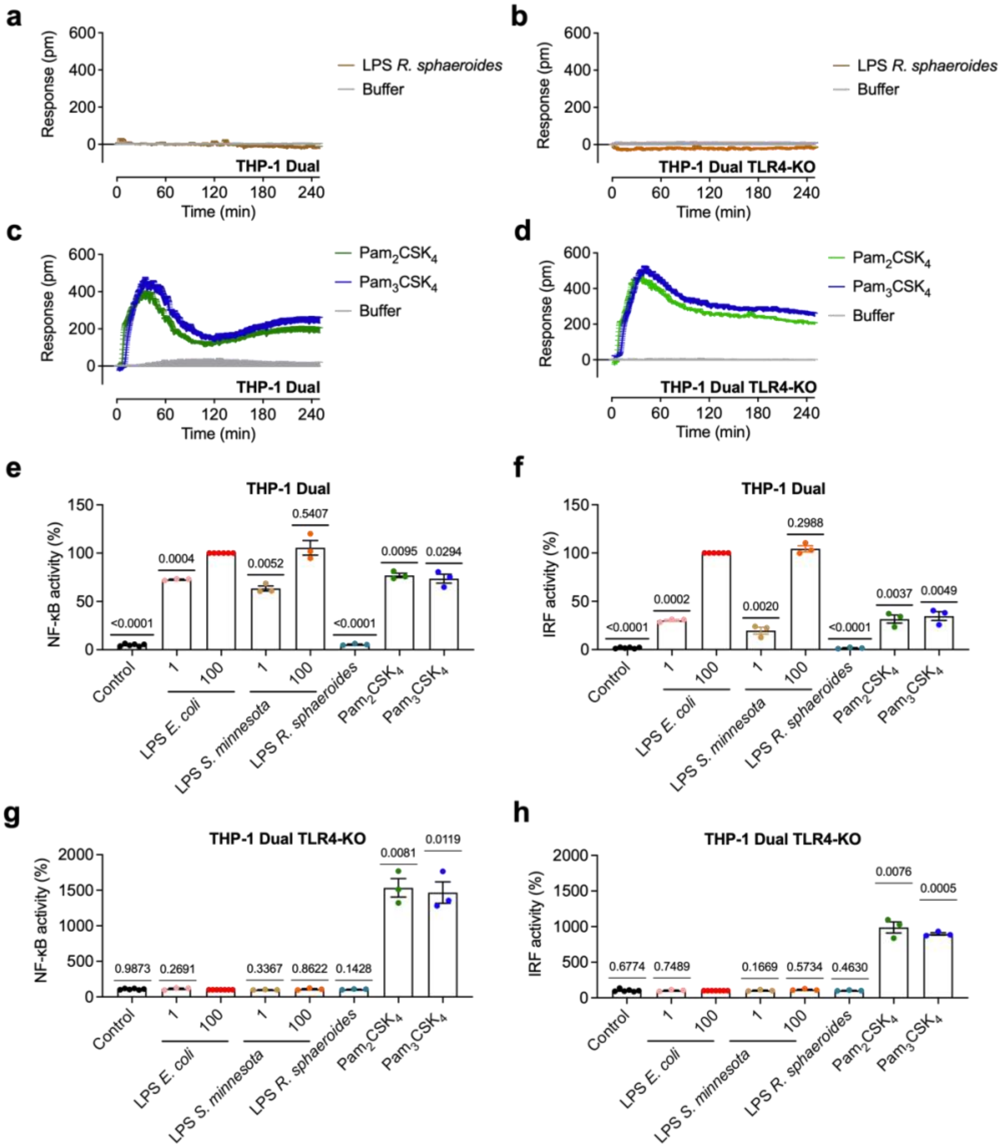

Supplementary Figure 9. THP-1 Dual TLR4-KO cells do not respond to LPS but TLR2 ligands. a-d THP-1 TLR4/MD-2/CD14 (THP-1 Dual) (**a, c**) or THP-1 Dual KO-TLR4/MD-2/CD14 (THP-1 Dual TLR4-KO) (**b**, **d**) cells were stimulated with LPS *R. sphaeroides* (1000 ng/ml) (**a**, **b**), Pam_3CSK_4 (1000 ng/ml) or Pam_2CSK_4 (1000 ng/ml) (**c**, **d**). Baseline-corrected DMR recordings are mean + SEM and representative of three biologically independent experiments. **e**-**h** THP-1 TLR4/MD-2/CD14 (THP-1 Dual) (**e**, **f**) or THP-1 Dual KO-TLR4/MD-2/CD14 (THP-1 Dual TLR4-KO) cells (**g**, **h**) were incubated for 24 h with LPS *E. coli* (1 and 100 ng/ml), LPS *S. minnesota* (1 and 100 ng/ml), or LPS *R. spharoides* (100 ng/ml). The TLR2 agonists Pam_3CSK_4 (1000 ng/ml) and Pam_2CSK_4 (1000 ng/ml) served as controls. After overnight incubation, activation of NF-κB was assessed by measuring the activity of SEAP in the supernatant using Quanti-Blue (**e**, **g**) or the IRF response (**f**, **h**) was assessed by measuring the activity of lucia luciferase in the supernatant using QUANTI-Luc. Data are normalized to LPS *E. coli* (100 ng/ml) and shown as mean ± SEM (*n* = 3 biological replicates except control and LPS *E. coli* 100 ng/ml, *n* = 6). One-sample *t* test against 100% (**e**-**h**). Source data are provided as a Source Data file.

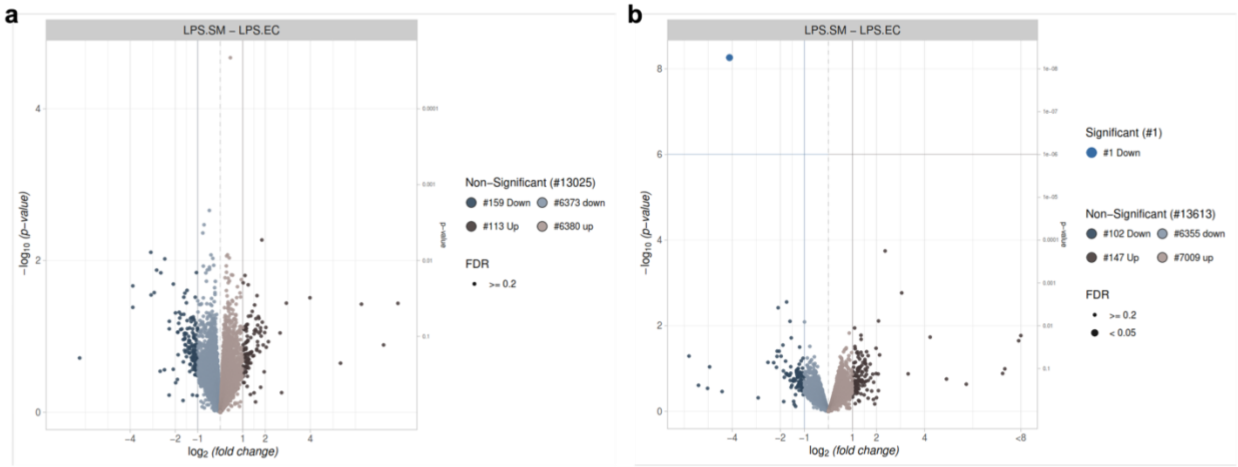

Supplementary Figure 10. Volcano plots show differential expression of LPS-induced genes in HEK293 TLR4/MD-2/CD14 cells and THP-1 macrophages. a, **b** The volcano plots of LPS *S. minnesota* vs. LPS *E. coli* in HEK293 TLR4/MD-2/CD14 cells (**a**) or THP-1 macrophages (**b**). For visualization, the package ggplot2 was used.

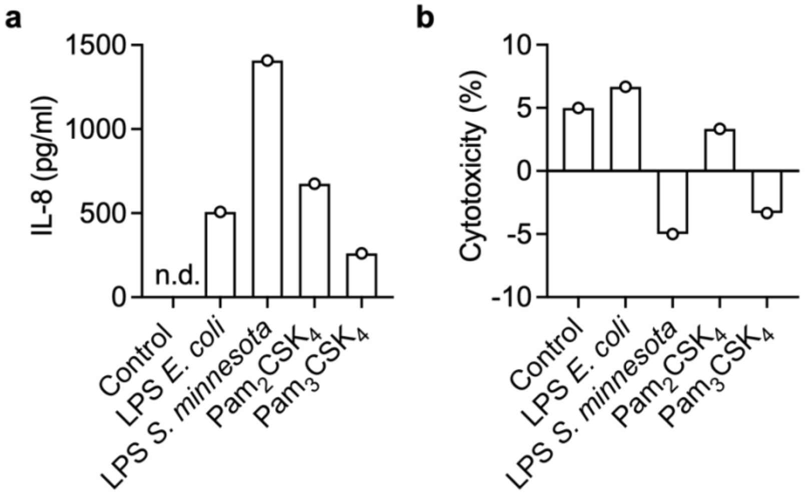

Supplementary Figure 11. TLR4 and TLR2 ligands trigger IL-8 release in primary monocytes.
a, **b** IL-8 secretion from primary monocytes (**a**) or cytotoxicity effect (**b**) following stimulation of the cells with LPS *E. coli* (10 ng/ml), LPS *S. minnesota* (10 ng/ml), Pam_2CSK_4 (1 µg/ml) or Pam_3CSK_4 (1 µg/ml) for 4 h. Cell-free supernatants were analyzed for IL-8 concentration by ELISA (**a**) and LDH release (**b**). Results are expressed as % of maximal LDH release. Representative results of one donor are shown. Source data are provided as a Source Data file.

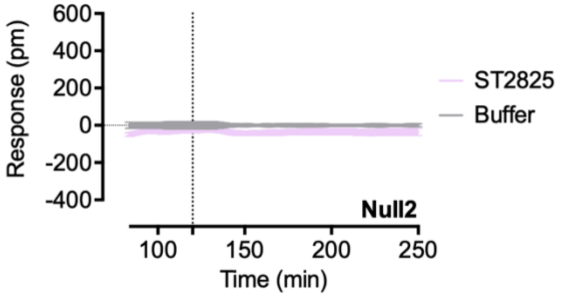

Supplementary Figure 12. MyD88 inhibitor ST2825 shows no DMR signals in HEK293 cells. HEK293 control cells lacking TLR4 (Null2) incubated with the MyD88 inhibitor ST2825 (10 µM). The dashed line indicates the preincubation time (120 min) of the inhibitor. Baseline-corrected DMR recordings are mean + SEM and representative of three biologically independent experiments. Source data are provided as a Source Data file.

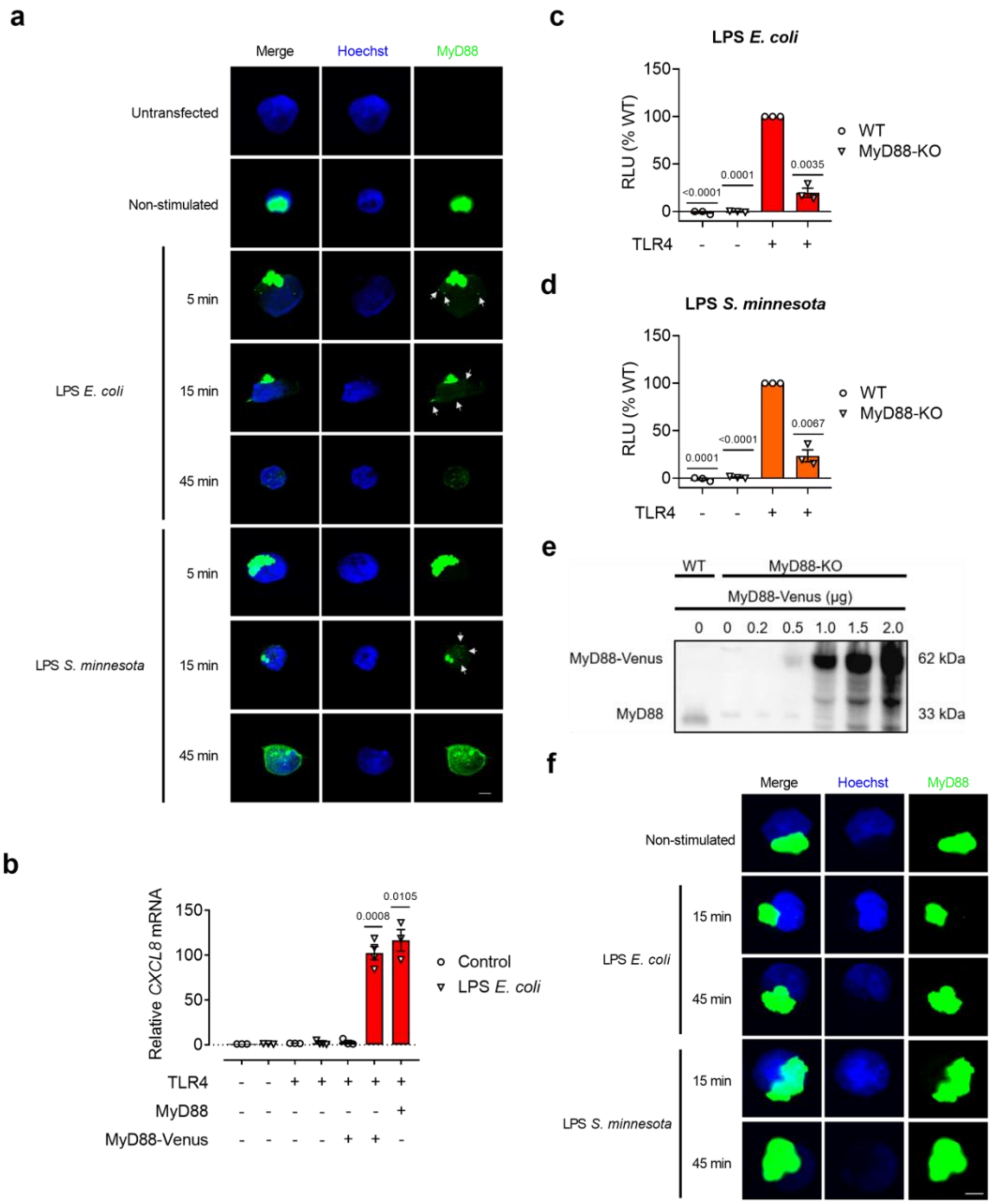

Supplementary Figure 13. MyD88 localization and functional analysis in HEK293 cells after LPS stimulation. a Immunofluorescence microscopy for localization experiments of MyD88 (green) in transfected HEK293 TLR4/MD-2/CD14 cells before and after stimulation with LPS *E. coli* and LPS *S. minnesota* (100 ng/ml) for 5 min, 15 min or 45 min. Cells are transfected with 200 ng MyD88-Venus construct and counterstained with the nuclear probe Hoechst (blue). Scale bar, 5 µm. Images are representative of three biologically independent experiments. **b** Total RNA was extracted from HEK293 MyD88-KO cells after stimulation for 4 h with LPS *E. coli* (100 ng/ml). *CXCL8* mRNA was measured by qRT-PCR and normalized to the expression of the housekeeping gene *GAPDH.* Data are mean ± SEM. n = 3 biologically independent experiments except for LPS *E. coli* + TLR4 and LPS *E. coli* + TLR4 + MyD88-Venus, n = 4). One-sample t test against 1. **c**, **d**

HEK293 WT or HEK293 MyD88-KO cells were transiently transfected with reporter constructs for ELAM luciferase, Renilla luciferase, MD-2, CD14 and with or without wild-type hTLR4. Cells were stimulated for 6 h with LPS *E. coli* (100 ng/ml) (**c**) or LPS *S. minnesota* (100 ng/ml) (**d**). Luciferase activity is expressed as percent of cells transfected with wild-type hTLR4 and stimulated with corresponding LPS concentrations (control, 100% activity). RLU, relative luciferase unit. Data are mean ± SEM (n = 3 biological replicates). One-sample t test against 100%. **e** Western blot analysis of the endogenous expression levels of MyD88 in HEK293 cells as well as MyD88-Venus after transfection in HEK293 MyD88-KO cells. Data are representative of three biologically independent experiments. Uncropped version of blot is provided in Supplementary Fig. 16. **f** Immunofluorescence microscopy for localization experiments of MyD88 (green) in transfected HEK293 MyD88-KO cells without TLR4 before and after stimulation with LPS *E. coli* and LPS *S. minnesota* (100 ng/ml) for 15 min or 45 min. Cells are transfected with 500 ng MyD88-Venus construct and counterstained with the nuclear probe Hoechst (blue). Scale bar, 5 µm. Images are representative of three biologically independent experiments. Source data are provided as a Source Data file.

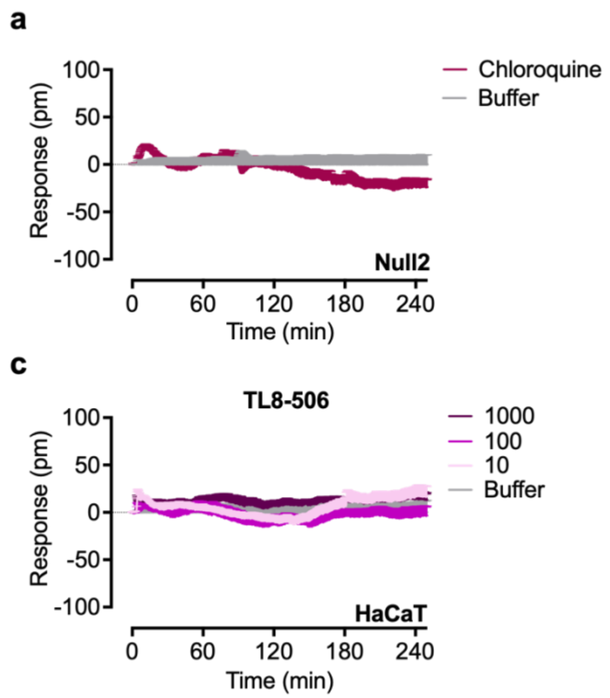

Supplementary Figure 14. Optical biosensor assay shows no DMR signals in control cells in the presence of TLR3 or TLR8 ligands. a Control HEK293 cells stimulated with chloroquine (15 µM). **b** HEK293 TLR8 reporter cells stimulated with the TLR8 inhibitor CU-CPT9a (1 µM). **c**, **d** HaCaT (**c**) and control HEK293 cells (Null2) (**d**) stimulated with the indicated concentrations (ng/ml) of the TLR8 agonist TL8-506. Baseline-corrected DMR recordings are mean + SEM and representative of three biologically independent experiments. Source data are provided as a Source Data file.

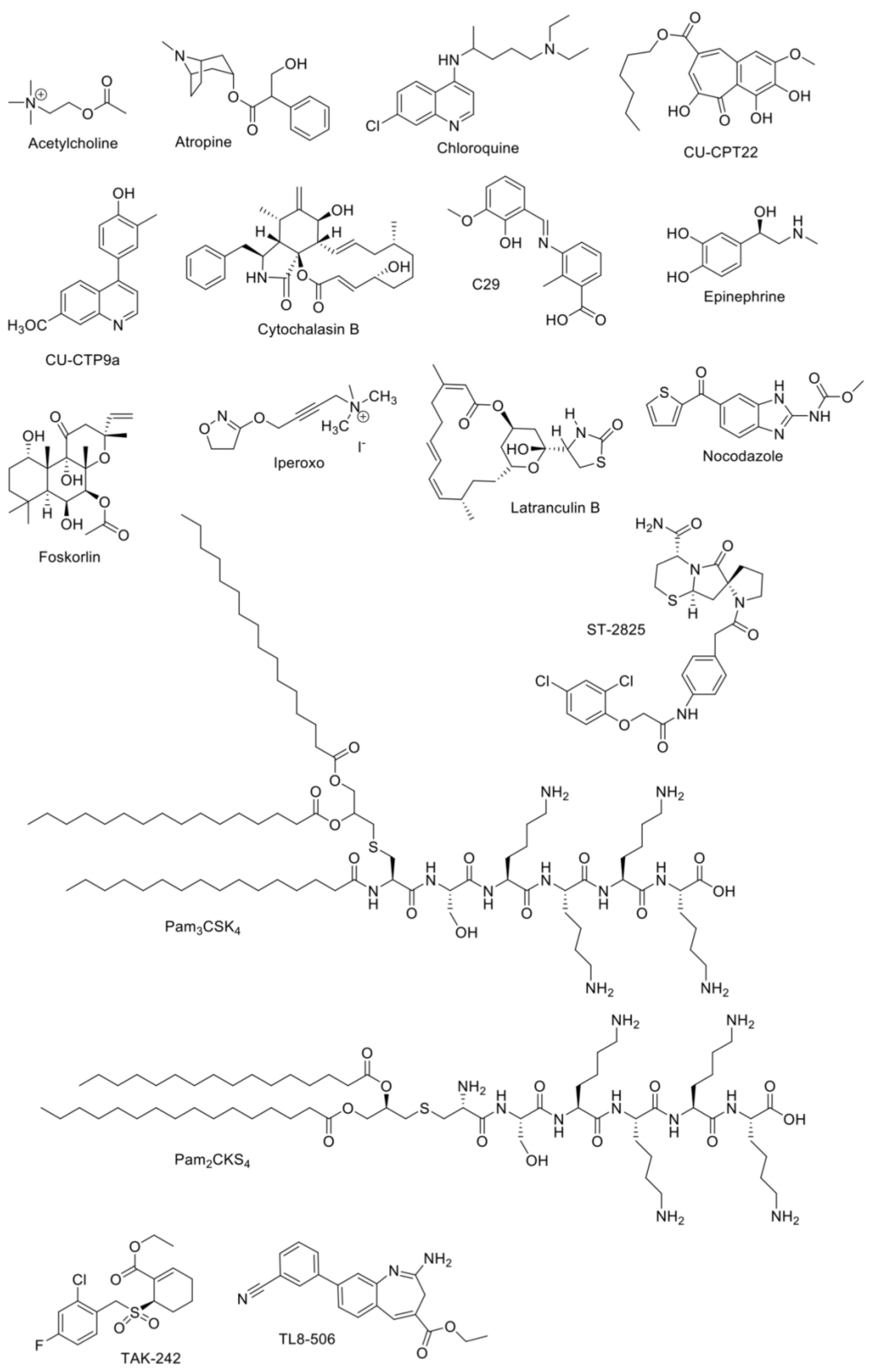

Supplementary Figure 15. Chemical structures of ligands and inhibitors.

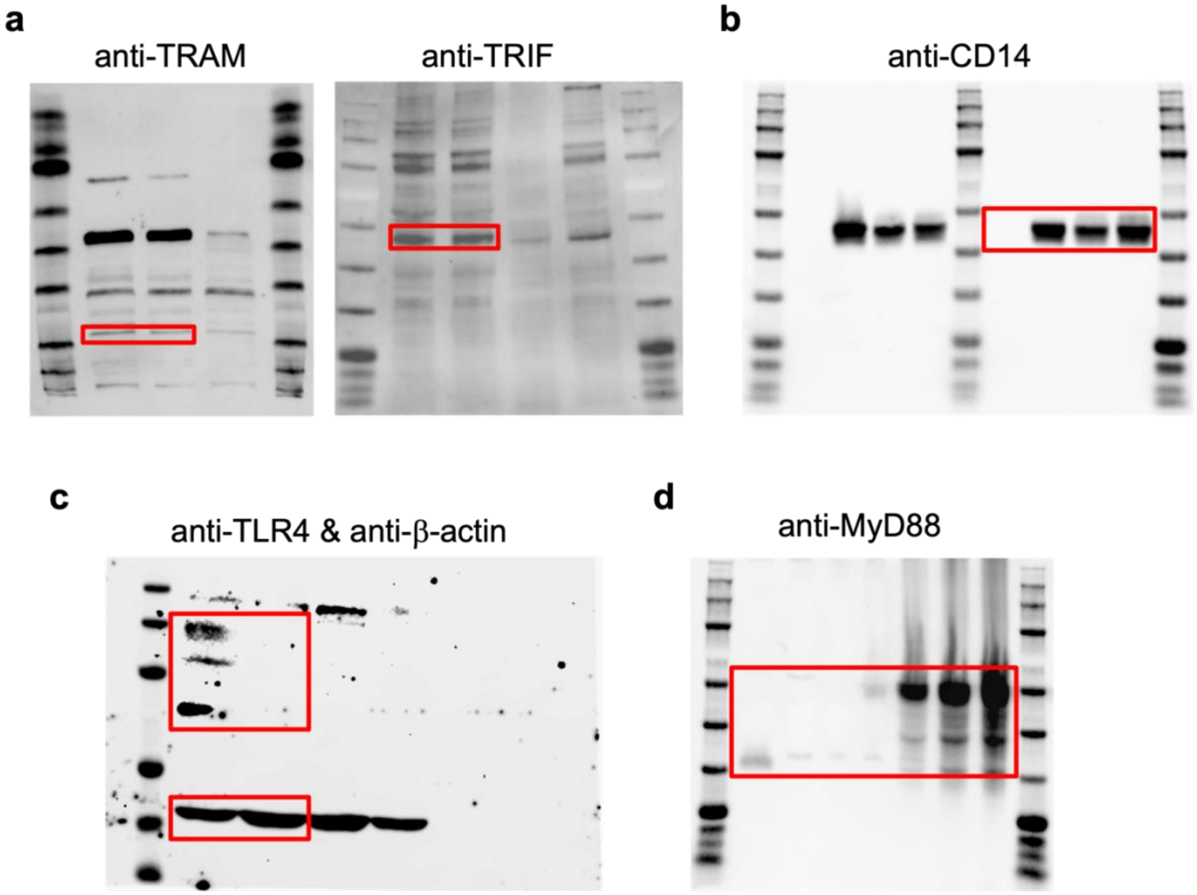

Supplementary Figure 16. Whole uncropped images of the original western blots. a Complete western blots of TRAM and TRIF shown in Supplemental Fig. 2. **b** Complete western blot of CD14 shown in Supplemental Fig. 7. **c** Complete western blot of TLR4 and β-actin shown in Supplemental Fig. 8a. **d** Complete western blot of MyD88 shown in Supplemental Fig. 13.

Supplementary References

1. Sforza, S. *et al.* Determination of fatty acid positions in native lipid A by positive and negative electrospray ionization mass spectrometry. *J. Mass Spectrom.* **39,** 378–383; 10.1002/jms.598 (2004).

2. Qureshi, N., Mascagni, P., Ribi, E. & Takayama, K. Monophosphoryl lipid A obtained from lipopolysaccharides of Salmonella minnesota R595. Purification of the dimethyl derivative by high performance liquid chromatography and complete structural determination. *J. Bio. Chem.* **260,** 5271–5278 (1985).

3. Irvine, K. L. *et al.* Identification of key residues that confer Rhodobacter sphaeroides LPS activity at horse TLR4/MD-2. *PloS one* **9,** e98776; 10.1371/journal.pone.0098776 (2014).

4. Alexander, C. & Rietschel, E. T. Bacterial lipopolysaccharides and innate immunity. *J. Endotoxin Res.* **7,** 167–202 (2001).
